# Decreased Interhemispheric Functional Connectivity and Its Associations with Clinical Correlates following Traumatic Brain Injury

**DOI:** 10.1155/2022/3408660

**Published:** 2022-04-07

**Authors:** Yu Song, Xi Han, Gaoyi Li, Wusong Tong, Mingxia Fan, Xianzhen Chen, Jia Yin, Songyu Chen, Jingrong Huang, Dongming Gao, Liang Gao, Yan Dong

**Affiliations:** ^1^Department of Neurosurgery, Shanghai Tenth People's Hospital, Tongji University School of Medicine, Shanghai, China; ^2^Department of Neurosurgery, Shanghai Hushan Hospital, Fudan University, Shanghai, China; ^3^Department of Neurosurgery, People's Hospital of Putuo District, Tongji University School of Medicine, Shanghai, China; ^4^Department of Neurosurgery, Shanghai Pudong New Area People's Hospital, Shanghai, China; ^5^Shanghai Key Laboratory of Magnetic Resonance, East China Normal University, Shanghai, China; ^6^Psychology Honors Program, University of California, San Diego, USA; ^7^Shanghai Tenth People's Hospital Clinical Medicine Scientific and Technical Innovation Park, Shanghai, China

## Abstract

**Objective:**

To explore the interhemispheric functional coordination following traumatic brain injury (TBI) and its association with posttraumatic anxiety and depressive symptoms.

**Methods:**

This was a combination of a retrospective cohort study and a cross-sectional observational study. We investigated the functional coordination between hemispheres by voxel-mirrored homotopic connectivity (VMHC). Grey matter volumes were examined by voxel-based morphometry (VBM), and microstructural integrity of the corpus callosum (CC) was assessed by diffusion tension imaging (DTI). The anxiety and depressive symptoms were evaluated with the Hospital Anxiety and Depression Scale.

**Results:**

The VMHC values of the bilateral middle temporal gyrus (MTG) and orbital middle frontal gyrus (MFG) were significantly decreased in TBI patients versus the healthy controls. Weakened homotopic functional connectivity (FC) in the bilateral orbital MFG is moderate positively correlated with anxiety and depressive symptoms. The white matter integrity in the CC was extensively reduced in TBI patients. In the receiver operating characteristic analysis, the VMHC value of the orbital MFG could distinguish TBI from HC with an area under the curve of 0.939 (sensitivity of 1 and specificity of 0.867).

**Conclusion:**

TBI disrupts the interhemispheric functional and structural connection, which is correlated with posttraumatic mood disorders. These findings may serve as a clinical indicator for diagnosis.

## 1. Introduction

Patients of all-severity traumatic brain injury (TBI) can be suffering from long-term mood disorders [1–3]. In the first year after TBI, the possibilities of diagnosing anxiety disorder and depression are around 21% and 17%, which might keep increasing over time, and such long-term mood disorders would negatively affect their quality of life [4]. In spite of considerable advances which have been made to explore the potential mechanisms involved in posttraumatic neuropsychiatric disorders, the exact neurobiological basis of anxiety and depressive disorders following TBI remains to be fully elucidated.

Robust homotopic resting-state functional connectivity is a fundamental characteristic of the intrinsic functional architecture of the brain [5, 6]. Zuo et al. developed voxel-mirrored homotopic connectivity (VMHC), a method exploring the interhemispheric functional connectivity in a voxel-wise manner [7]. The observations that homotopic resting-state functional connectivity was regionally specific and sex-related in the life span developmental trajectories have inspired great interest in the implication of functional homotopy in neurodegenerative and psychiatric disorders [7]. In the populations with depression, disrupted interhemispheric functional connectivity was observed within multiple brain regions, with the cognitive control network (CCN) and the default mode network (DMN) being the most commonly identified, which was correlated with clinical features in depression. Recently, disrupted homotopic functional connectivity has been observed in generalized anxiety disorder [8]. The corpus callosum (CC), the major white matter tract of the cerebral commissural system, plays a vital role in the structural interactions between bilateral hemispheres. Existing data confirmed that abnormalities of CC may influence interhemispheric functional communications and participate in the pathology of diseased conditions [8–10].

In terms of homotopic functional connectivity following TBI, two studies have been reported. One previous traumatic axonal injury (TAI) patient study found that TAI contributes to the VMHC decreases in several brain regions, which involve various neuronetworks [11]. According to the other longitudinal study that examined both dynamic interhemispheric structures and functional connectivity (FC) in mild traumatic brain injury (mTBI) patients, the impairment of certain regions in CC corresponded well with the FC decrease of specific brain regions. It also suggested that the poor executive function in mild TBI patients is found to be related to the VMHC decline of the dorsolateral prefrontal cortex [12]. Although former studies have discussed extensive disturbances of interhemispheric connectivity, the neurobiological basis of the interhemispheric functional asynchronization and its implications in posttraumatic mood disorders remains unclear.

In this study, we investigated the functional coordination between hemispheres by VMHC, examined the grey matter volumes within the regions with altered VMHC by voxel-based morphometry (VBM), and assessed microstructural CC integrity by diffusion tension imaging (DTI). We aimed to explore the disturbance of interhemispheric functional coordination in TBI individuals and their associations with posttraumatic anxiety and depressive symptoms. Furthermore, we probed whether the disrupted interhemispheric functional coordination resulted from structural alterations.

## 2. Materials and Methods

### 2.1. Study Design

Our research consisted of two study designs: retrospective cohort study and cross-sectional observational study. First, we retrospectively collected the data of TBI patients and explored disturbances of the functional coordination between hemispheres following TBI by VMHC. This was a retrospective cohort study. Second, we further analyzed the correlation between the changed interhemispheric functional connectivity and posttraumatic anxiety and depressive symptoms. The second experiment was considered as a cross-sectional observational study.

### 2.2. Subjects

The present study included 33 TBI patients and 23 healthy controls (HC). TBI patients admitted to the Pudong New Area People's Hospital in Shanghai from December 2016 to March 2019 were included if they met the criteria as follows: (1) older than 18 years; (2) with the first TBI onset; (3) with the positive finding on cranial CT on admission (including contusion, intracranial hematoma, epidural/subdural hemorrhage, and traumatic subarachnoid hemorrhage). Patients with neurological or psychiatric disorders before TBI were excluded. The HCs were recruited matching for age, gender, and education with the TBI group. There were no contraindications to MRI in all participants. This study was approved by the ethics committee of the Pudong New Area People's Hospital. Patients were deemed to have a capacity/ability to consent if they had the ability to communicate a reasoned choice regarding participation and the ability to understand relevant information about the study, including the purpose of the study, the procedures to which the participants would be exposed, the benefits or risks to participants, and the right to decide whether or not to participate in the research study. The reporting of our study met STROBE guidelines for retrospective cohort and cross-sectional studies (Supplementary Checklist (available here)).

### 2.3. Clinical Data Collection

Demographic- and TBI-related parameters were extracted from the electronic database. The initial severity of TBI was assessed within the first 24 hours after injury using the Glasgow Coma Scale (GCS). Cranial CT on admission was diagnosed by neuroradiologists. At the follow-up visits, Glasgow Outcome Scale-extended (GOS-E) was used to evaluate the condition of the patients. Traumatic anxiety and depression were examined by trained physicians using the Hospital Anxiety and Depression Scale (HADS), which was proved to have high reliability and validity in previous TBI studies [13, 14].

### 2.4. MRI Data Acquisition and Processing

MRI data was acquired by Siemens Prisma 3.0 Tesla MRI system (Prisma, Siemens, Erlangen, Germany) at the follow-up visits the same day after neuropsychiatric assessment. The sequences included rs-fMRI, DTI, and high-resolution T1-weighted magnetization prepared rapid acquisition gradient echo (MPRAGE) sequences. Participants lied supine in the MRI scanner, with a foam pad to prevent head motion and earplugs to reduce noise. Patients were told to close their eyes, stay awake, and avoid thinking about anything in particular during the test time. The parameters were set as follows. For rs-fMRI data, 240 contiguous EPI functional volumes were obtained, with 33 axial slices, slice thickness = 3.5 mm, gap = 0.7 mm, time of repetition (TR) = 2000 ms, time of echo (TE) = 30 ms, flip angle = 90°, field of view (FOV) = 224 mm × 224 mm, matrix size = 64 × 64; and voxel size = 3.5 × 3.5 × 3.5 mm^3^. DTI data was acquired for 30 gradient directions, 1 baseline (*b* = 0) image, *b* = 1000 s/mm^2^, TR = 10100 ms, TE = 92 ms, FOV = 256 mm × 256 mm, 75 axial slices, and voxel size = 2.0 × 2.0 × 2.0 mm^3^. For high-resolution T1-weighted MPRAGE data, 192 sagittal slices were obtained, thickness = 1 mm, gap = 0.5 mm, TR = 2530 ms, TE = 2.98 ms, flip angle = 7°, FOV = 256 mm × 256 mm, matrix size = 256 × 256, and voxel size = 1.0 × 1.0 × 1.0 mm^3^.

Functional MRI data analysis was performed using Data Processing Assistant for Resting-State fMRI (DPARSF) toolbox [15]. The following steps were applied: (1) converting the format, from DICOM to NIFTI; (2) discarding the first ten volumes of each raw rs-fMRI data and performing slice-timing correction; (3) realigning the time series of images; (4) coregistrating T1-weighted images to the mean functional image and then segmenting into grey matter (GM), white matter (WM), and cerebrospinal fluid (CSF); and (5) transforming from individual native space to Montreal Neurological Institute (MNI) space by using the Diffeomorphic Anatomical Registration Through Exponentiated Lie algebra (DARTEL) tool. Nuisance regressions included the Friston 24-parameter model, mean frame-wise displacement (FD, derived from Jenkinson's relative root mean square algorithm [16]), cerebral spinal fluid and white-matter signals, and scrubbing (removing time points with FD > 0.2 mm). Spatial smoothing was performed using a 4-mm full-width half-maximum (FWHM) Gaussian kernel. In addition, a linear trend was included as a regressor to account for drifts in the blood oxygen level-dependent level (BOLD) signal. Temporal bandpass filtering (0.01-0.1 Hz) was performed to reduce the effect of low-frequency drift and high-frequency noise.

For VMHC calculation, the Pearson correlation coefficient of each pair of symmetrical voxel BOLD signals between hemispheres in the brain was first calculated. Then, the coefficients were Fisher-*Z* transformed to make sure normal distribution for further statistical analysis.

Structural analyses were performed using SPM12 (http://www.fil.ion/. http://ucl.ac.uk/spm/) and CAT12 (CAT12; http://dbm.neuro.unijena.de/cat12/). After correcting for bias field in homogeneities, the T1-weighted image was segmented into different components using the DARTEL algorithm and transformed into MNI space [17]. Voxel-based morphometry (VBM) analysis was used to calculate the brain region volume and total intracranial volume (TIV) was calculated as the sum of the grey matter, white matter, and cerebrospinal fluid volumes.

DTI data were analyzed using Functional Magnetic Resonance Imaging of the Brain Software Library (FSL 5.0, Oxford Centre). In brief, the b0 image was used for brain extraction. Then, the motion movement and eddy current correction were performed. Finally, the fractional anisotropy (FA) was fitted in each voxel. The average value of the whole region of interest (ROI) was calculated.

### 2.5. Sample Size Estimates

Since our research was a combination of a retrospective cohort study and a cross-sectional observational study, we did not calculate the sample size. On the other hand, the sample size of 20-30 participants is commonly used in the resting-state fMRI studies. According to the previous studies [11, 12], we set the sample size as not less than 20. We recruited all participants according to the inclusion and exclusion criteria set for the study. Finally, 33 TBI participants and 23 healthy controls were included.

### 2.6. Statistical Analyses

Statistical analyses were conducted using R (Version 4.0.0 R Foundation for Statistical Computing, Vienna, Austria) and DPARSF. Continuous variables were described using median and interquartile range (IQR), and categorical variables were summarized using frequencies. The group comparisons were analyzed using the Mann–Whitney *U*-test for continuous variables and the Chi-squared test for categorical variables.

Group comparisons for VMHC were analyzed using DPARSF with a two-sample *t*-test, controlling for age, gender, education, mean FD, and grey matter density. Correction for multiple comparisons was performed by Gaussian random field correction (GRF) (significance level was set at voxel *p*-value <0.001 and cluster *p*-value <0.05). Relations of VMHC with psychological parameters were examined by partial correlations, controlling for age, gender, education, and TIV. Multiple linear regression analysis was used to study the difference in grey matter volume and integrity of CC between the two groups, adjusting for age, gender, education, and TIV. Results were considered significant if the *p*-value was less than 0.05.

Receiver operating characteristic (ROC) analysis was used to evaluate the predictive power of the altered VMHC distinguishing the TBI patient group from the HC group. The area under curve (AUC) sensitivity, specificity, and cutoff were calculated.

## 3. Results

### 3.1. Demographic and Clinical Characteristics

Three TBI patients and one HC were excluded due to head motion on the rs-fMRI scanning. The final study cohort contains thirty TBI patients and twenty-two HCs. There were no significant differences between the TBI group and healthy controls in terms of gender (*p* = 0.946), age (*p* = 0.565), education years (*p* = 0.795), or head motion (*p* = 0.745). The median time since injury was six months. All the TBI patients showed CT positive findings on admission and among them twenty-three patients presented focal lesions on follow-up MRI scanning, mainly located in the prefrontal and temporal lobes near the skull base. The TBI group showed significant abnormalities in the neuropsychological assessment, showing severe anxiety (*p* = 0.002) and depression (*p* = 0.002) versus the HC group (Table 1).

### 3.2. Regional Variation in Interhemispheric Functional Connectivity following TBI

**Figure 1:**
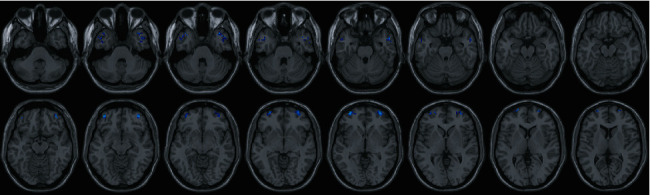
Regional variation of VMHC between TBI patients and the healthy controls. Relative to healthy controls, the TBI patients had significantly decreased VMHC values (blue) in 4 clusters, including the middle temporal gyrus and orbital middle frontal gyrus on both sides.

We explored the regional alteration in interhemispheric functional connectivity using VMHC. Compared with the healthy controls, the TBI patients had significantly decreased VMHC values in 4 clusters, including the middle temporal gyrus (MTG) and orbital middle frontal gyrus (MFG) on both sides (Table 2). No brain regions showed increased VMHC in the TBI group (Figure 1).

### 3.3. Association of VMHC with Anxiety and Depressive Symptoms following TBI

**Figure 2:**
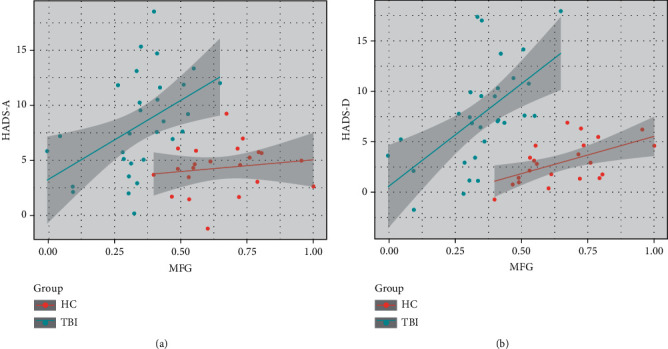
Partial correlation analyses in the TBI group and healthy controls. A and B showed the partial correlations between the VMHC value of the orbital middle frontal gyrus and HADS anxiety and depression scores, respectively. MFG: middle frontal gyrus.

The TBI patients showed significantly moderate positive correlations between VMHC of the bilateral orbital MFG and mood disorder, especially with the depression status after being adjusted for age, gender, education, and TIV (rho = 0.578, *p* = 0.002). In the HC group, there was also a significant moderate positive correlation between bilateral orbital MFG and depression score after adjusting for age, sex, education, and TIV (rho = 0.555, *p* = 0.017). No significant correlations were observed between the MTG and neuropsychological measures (Table 3 and Figure 2).

### 3.4. Diagnostic Value of Changed VMHC in Differentiating TBI from HC

**Figure 3:**
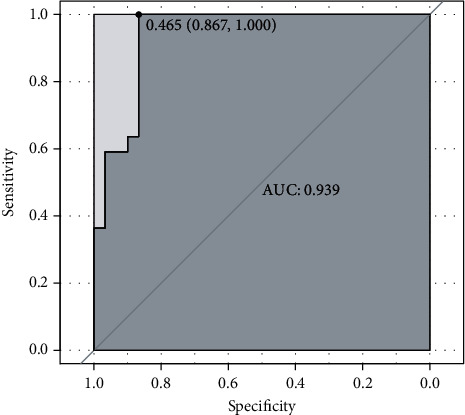
The diagnostic value of the decreased VMHC in distinguishing the TBI patients from the healthy controls. AUC: area under the curve.

ROC analysis demonstrated that changed VMHC values could distinguish TBI from HC. Optimal performance was achieved in the VMHC of the orbital MFG at an AUC of 0.939 (sensitivity of 1 and specificity of 0.867; Figure 3).

### 3.5. Regional Variation in Grey Matter Volume following TBI

Grey matter volume was assessed in the brain regions that showed significant differences in VMHC analysis. No significant change in the bilateral MFG grey matter volume between the two groups after adjusting for age, gender, education, and TIV, while the left side of MTG grey matter volume in the TBI group was significantly lower than that of the healthy control group at a threshold of *p* < 0.05 (Table 4).

### 3.6. The Integrity of White Matter Connectivity in the Corpus Callosum following TBI

**Figure 4:**
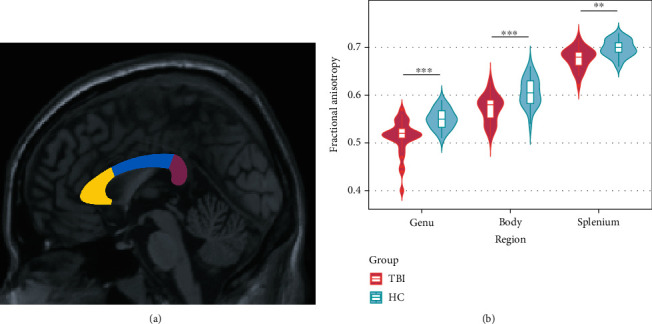
Comparisons on FA values in each subregion of the corpus callosum between TBI and HC. (a) Three parts of the corpus callosum. (b) Univariable analysis of the three subregions between the two groups.

FA value in three subregions of CC was also analyzed. The TBI group showed significantly white matter disruption in all three subregions both before and after adjusting for age, gender, education, and TIV (Table 5 and Figure 4).

## 4. Discussion

As the most commonly observed psychiatric disorders following TBI, anxiety and depression may severely impact patients' life in many aspects. However, the specific brain location associated with such disorders still needs investigation. In the current study, we found the VMHC values in the bilateral MTG and orbital MFG were significantly decreased in TBI patients. Weakened homotopic FC in the orbital MFG was found to have a moderate positive correlation with anxiety and depressive symptoms, and such alteration of homotopic connectivity depends on the white matter integrity.

CC is one of the major commissural fibers playing an important role in interhemispheric interactions, and it facilitates bilateral sensory integration and higher cognitive functions [9, 18]. Patients with corpus callosotomy exhibited severe, nonreversible interhemispheric connectivity disruption in several brain regions [19]. In major depressive disorder (MDD), fibers crossing genu, rostrum, and splenium were also reported anatomically damaged, accompanied by damage to the grey matter in the corresponding areas [20]. The injury of the middle part of CC may serve as an imaging biomarker for generative anxiety disorder (GAD) patients [8]. In TBI patients, CC is also one of the most vulnerable structures, even if there is no visible injury on admission CT scan and results in many neuropsychiatric disorders [12, 21–23]. In our study, FA decrease in all three subregions of the CC. Specific brain regions interhemispheric connectivity correlated with specific CC fibers; thus, extensive CC damage seemed to result in VMHC decreasing in all brain regions. However, in our cohort, functional connectivity between bilateral frontal lobes and bilateral temporal lobes showed more sensitivity to CC structural damage. On one hand, both two brain regions were adjacent to the injury sites caused by TBI. Injury to the cortex decreased interhemispheric connectivity. On the other hand, Roland and colleagues pointed that some other regions' interhemispheric connectivity does not pass through the CC [19]. These two reasons may be explained why only VMHC in frontal and temporal lobes decreased during widespread deterioration in CC in our study.

The frontal lobe and temporal lobe are two main direct targets of TBI [24, 25]. A previous meta-analysis demonstrated frontal regions and temporal regions were the most vulnerable areas during mTBI [26], which was consistent with our lesion map. Previous traumatic axonal injury analysis also mentioned that the decreased FC in the inferior frontal gyrus was associated with emotional disorders [11]. In the present study, we further narrowed the damage location of the frontal lobe to the orbital MFG. The orbital MFG is in the prefrontal cortex region of the frontal lobes. Altered VMHC values in the orbital MFG bilaterally were found in peripheral facial neuritis [27], obsessive-compulsive disorder [28], internet gaming disorder [29], and epilepsy [30]. While in TBI patients, our data suggested that decreased VMHC in the orbital MFG can distinguish chronic TBI from the healthy controls with AUC up to 93.9%.

The critical role of the orbitofrontal cortex in the emotion and reward system is indicated by its complex connections with other emotion-related areas like the thalamus [31]. Further, damaging the orbital MFG can produce depression as the reward system breaks down [32]. The frequent concurrences of depression and anxiety often occur together, of which the incidence of comorbidities can be up to 60% [33], which might be explained by their similar biological mechanism [34]. GAD [35–37], MDD [38], and posttraumatic emotional dysfunction were reported having a shared deficit area—orbitofrontal cortex, which indicates that these kinds of emotional disorders may have a similar diagnostic method and apply the same treatments.

In our cohort, TBI not only led to the decrease of VMHC in the orbital MFG but also contribute to the alteration of the correlativity between VMHC and clinical symptoms. Specifically, the decreased VMHC value in the orbital MFG is moderately positively correlated with the anxiety and depression score. The result is somewhat abnormal, for TBI patients suffered decreased VMHC in the orbital MFG compared to HCs. We speculated that the correlation between them is nonlinear, thus further studies are needed to explore the specific correlation. In addition, some studies suggest that the milder the TBI is, the more severe neuropsychiatric symptoms are reported, which may be another explanation for the anomalous correlation [39].

Moreover, similar to previous findings, the bilateral MTG also showed significant destruction in TBI patients [11, 40]. However, we failed to find the correlation between VMHC and neuropsychiatric disorders. Another focus that future research could focus on is whether bilateral temporal lobe FC injury is associated with memory impairment, for it is the center of memory function [40].

However, several limitations in this study need to be mentioned. Firstly, the sample size was relatively small in both groups, which may limit statistical power. Secondly, the severity of our recruited TBI patients at admission was largely various across different levels. GCS was used to assess the severity of comatose patients, but it might not accurately depict intracranial conditions. But still, it has the potential to impact our results. Thirdly, this was a combination of retrospective cohort and cross-sectional studies that failed to demonstrate changes in brain function during the time period of recovery. Further studies should include more patients with careful imaging stratification (type of injury, severity of injury, site of injury, etc.) and perform longitudinal follow-up observation to validate our findings.

## 5. Conclusion

The present study found that participants with TBI showed deficits in VMHC between bilateral orbital MFG and MTG and structural damage in all three parts of CC compared to the healthy controls. Furthermore, HADS-A and HADS-D are moderate positively correlated with the change in VMHC in the orbital MFG. These findings demonstrated the important role of interhemispheric FC of the prefrontal lobe in the neuropathological mechanism of TBI. Knowing this specific location, the diagnosis of emotional dysfunction after TBI is easier and facilitates further treatment.

## Figures and Tables

**Table 1:** Demographic and clinical characteristics of the enrollment.

	Overall	TBI	HC	*p*-value
	(*n* = 52)	(*n* = 30)	(*n* = 22)	
Gender (%)				0.946
Male	34 (65.4)	19 (63.3)	15 (68.2)	
Female	18 (34.6)	11 (36.7)	7 (31.8)	
Age (median [IQR])	32.50 [25.75, 45.00]	34.50 [26.00, 45.75]	31.50 [25.00, 41.75]	0.565
Education (median [IQR])	9.00 [7.50, 12.00]	9.00 [6.00, 12.00]	9.00 [9.00, 11.25]	0.795
Time since injury (median [IQR])		6 [4.00, 11.00]		—
Cause of injury (%)				
Traffic accident		19 (63.33)		—
Fall		7 (23.33)		—
Struck by/against		4 (13.33)		—
GCS on admission (%)				
13-15		20 (66.67)		—
9-12		5 (16.67)		—
3-8		5 (16.67)		—
Loss of consciousness (%)				
Yes		19 (63.33)		—
No		9 (30.00)		—
Unknown		2 (6.67)		—
Intracerebral lesion on MRI scan (%)				
Yes		23 (76.67)		—
No		7 (23.33)		—
GOS-E (median [IQR])		6.50 [5.00,7.00]		—
Anxiety (median [IQR])	6.00 [3.75, 8.25]	7.00 [5.00, 12.00]	4.00 [3.00, 6.00]	**0.002**
Depression (median [IQR])	4.50 [2.00, 7.25]	6.50 [3.25, 10.00]	2.50 [1.00, 4.75]	**0.002**

TBI: traumatic brain injury; HC: healthy control; IQR: interquartile range; GCS: Glasgow coma scale; and GOS-E: Glasgow outcome scale-extended.

**Table 2:** Regions showing significant differences in VMHC between traumatic brain injury patients and healthy controls.

Regions	MNI coordinates			Number of voxels	Peak intensity
	*X*	*Y*	*Z*		
TBI < HC					
MFG	±33	57	-3	40	-5.8005
MTG	±39	18	-33	32	-4.317

VMHC: voxel-mirrored homotopic connectivity; TBI: traumatic brain injury; MNI: Montreal Neurological Institute; HC: healthy control; MFG: middle frontal gyrus; and MTG: middle temporal gyrus.

**Table 3:** Partial correlation between clinical assessment and VMHC∗.

Regions	HADS-A		HADS-D	
	Rho	*p*-value	Rho	*p*-value
TBI group(*n* = 30)				
MFG	0.471	**0.015**	0.587	**0.002**
MTG	0.064	0.757	0.208	0.308
HC group(*n* = 22)				
MFG	0.151	0.55	0.555	**0.017**
MTG	0.372	0.129	0.283	0.256

VMHC: voxel-mirrored homotopic connectivity; TBI: traumatic brain injury; HC: healthy control; HADS-A: hospital anxiety and depression scale anxiety; HADS-D: hospital anxiety and depression scale depression; MFG: middle frontal gyrus; MTG: middle temporal gyrus. ∗Adjusted for age, gender, education (years), and total intracranial volume.

**Table 4:** The difference of grey matter volume between traumatic brain injury patients and healthy controls.

Regions	Univariable analysis			Multivariable analysis∗	
	TBI (*n* = 30)	HC (*n* = 22)	*p*-value	*β*	*p*-value
MFG-L	17.02 [14.65, 18.28]	18.07 [16.02, 20.29]	0.067	0.904	0.123
MFG-R	16.77 [15.28, 18.84]	18.52 [16.65, 20.80]	**0.023**	0.982	0.114
MTG-L	12.35 [11.60, 13.74]	13.49 [12.73, 15.30]	**0.008**	1.028	**0.012**
MTG-R	13.26 [11.99, 14.91]	14.50 [12.92, 15.76]	0.054	0.371	0.282

TBI: traumatic brain injury; HC: healthy control; MFG: middle frontal gyrus; MTG: middle temporal gyrus. ∗Dependent variable: grey matter volume; Independent variable: group. Adjusted for age, gender, education (years), and total intracranial volume.

**Table 5:** Comparison of fractional anisotropy values in different regions of the corpus callosum between traumatic brain injury patients and healthy controls.

Region	Univariable analysis			Multivariable analysis∗	
	TBI (*n* = 30)	HC (*n* = 22)	*p*-value	*β*	*p*-value
Genu	0.52 [0.51, 0.53]	0.55 [0.53, 0.57]	**<0.001**	0.038	**<0.001**
Body	0.58 [0.55, 0.59]	0.60 [0.58, 0.63]	**<0.001**	0.034	**<0.001**
Splenium	0.68 [0.66, 0.69]	0.70 [0.69, 0.71]	**0.002**	0.021	**0.001**

TBI: traumatic brain injury; HC: healthy control. ∗Dependent variable: FA value; Independent variable: group. Adjusted for age, gender, education (years), and total intracranial volume.

## Data Availability

The datasets generated for this study are available on request from the corresponding author.

## Supplementary Materials

Supplementary 1 Supplement 1: the raw data of our research.

Supplementary 2 Supplement 2: STROBE checklist of our research.

## References

[1] Scheenen M. E., Spikman J. M., de Koning M. E. (2017). Patients "at risk" of suffering from persistent complaints after mild traumatic brain injury: the role of coping, mood disorders, and post-traumatic stress. *Journal of Neurotrauma*.

[2] Stern R. A., Daneshvar D. H., Baugh C. M. (2013). Clinical presentation of chronic traumatic encephalopathy. *Neurology*.

[3] Mez J., Daneshvar D. H., Kiernan P. T. (2017). Clinicopathological evaluation of chronic traumatic encephalopathy in players of American football. *JAMA*.

[4] Scholten A. C., Haagsma J. A., Cnossen M. C., Olff M., van Beeck E. F., Polinder S. (2016). Prevalence of and risk factors for anxiety and depressive disorders after traumatic brain injury: a systematic review. *Journal of Neurotrauma*.

[5] Salvador R., Martínez A., Pomarol-Clotet E. (2008). A simple view of the brain through a frequency-specific functional connectivity measure. *NeuroImage*.

[6] Stark D. E., Margulies D. S., Shehzad Z. E. (2008). Regional variation in interhemispheric coordination of intrinsic hemodynamic fluctuations. *The Journal of Neuroscience*.

[7] Khundrakpam B. S., Lewis J. D., Jeon S. (2010). Growing together and growing apart: regional and sex differences in the lifespan developmental trajectories of functional homotopy. *The Journal of Neuroscience*.

[8] Wang W., Peng Z., Wang X. (2019). Disrupted interhemispheric resting-state functional connectivity and structural connectivity in first-episode, treatment-naive generalized anxiety disorder. *Journal of Affective Disorders*.

[9] De Leon Reyes N. S., Bragg-Gonzalo L., Nieto M. (2020). Development and plasticity of the corpus callosum. *Development*.

[10] Graham N. S. N., Jolly A., Zimmerman K. (2020). Diffuse axonal injury predicts neurodegeneration after moderate-severe traumatic brain injury. *Brain*.

[11] Li J., Gao L., Xie K. (2017). Detection of functional homotopy in traumatic axonal injury. *European Radiology*.

[12] Wang Z., Zhang M., Sun C. (2021). Single mild traumatic brain injury deteriorates progressive interhemispheric functional and structural connectivity. *Journal of Neurotrauma*.

[13] Dahm J., Wong D., Ponsford J. (2013). Validity of the Depression Anxiety Stress Scales in assessing depression and anxiety following traumatic brain injury. *Journal of Affective Disorders*.

[14] McKenzie D. P., Downing M. G., Ponsford J. L. (2018). Key Hospital Anxiety and Depression Scale (HADS) items associated with DSM-IV depressive and anxiety disorder 12-months post traumatic brain injury. *Journal of Affective Disorders*.

[15] Yan C., Wang X., Zuo X., Zang Y. F. (2016). DPABI: data processing & analysis for (resting-state) brain imaging. *Neuroinformatics*.

[16] Yan C. G., Chen X., Li L. (2019). Reduced default mode network functional connectivity in patients with recurrent major depressive disorder. *Proceedings of the National Academy of Sciences of the United States of America*.

[17] Ashburner J. (2007). A fast diffeomorphic image registration algorithm. *NeuroImage*.

[18] Yuan J., Song X., Kuan E. (2020). The structural basis for interhemispheric functional connectivity: Evidence from individuals with agenesis of the corpus callosum. *NeuroImage Clinical*.

[19] Roland J. L., Snyder A. Z., Hacker C. D. (2017). On the role of the corpus callosum in interhemispheric functional connectivity in humans. *Proceedings of the National Academy of Sciences of the United States of America*.

[20] Lee S., Pyun S. B., Choi K. W., Tae W. S. (2020). Shape and volumetric differences in the corpus callosum between patients with major depressive disorder and healthy controls. *Psychiatry Investigation*.

[21] Olczak M., Poniatowski L., Kwiatkowska M., Samojłowicz D., Tarka S., Wierzba-Bobrowicz T. (2019). Immunolocalization of dynein, dynactin, and kinesin in the cerebral tissue as a possible supplemental diagnostic tool for traumatic brain injury in postmortem examination. *Folia Neuropathologica*.

[22] Mazwi N. L., Izzy S., Tan C. O. (2019). Traumatic microbleeds in the hippocampus and corpus callosum predict duration of posttraumatic amnesia. *The Journal of Head Trauma Rehabilitation*.

[23] Nakayama N., Okumura A., Shinoda J. (2006). Evidence for white matter disruption in traumatic brain injury without macroscopic lesions. *Journal of Neurology, Neurosurgery, and Psychiatry*.

[24] Wang X., Xie H., Cotton A. S. (2015). Early cortical thickness change after mild traumatic brain injury following motor vehicle collision. *Journal of Neurotrauma*.

[25] Lu L., Li F., Chen H. (2020). Functional connectivity dysfunction of insular subdivisions in cognitive impairment after acute mild traumatic brain injury. *Brain Imaging and Behavior*.

[26] Eierud C., Craddock R. C., Fletcher S. (2014). Neuroimaging after mild traumatic brain injury: Review and meta-analysis. *NeuroImage Clinical*.

[27] Ma J., Hua X. Y., Zheng M. X. (2020). Structural remodeling secondary to functional remodeling in advanced-stage peripheral facial neuritis. *Neurological Sciences*.

[28] Deng K., Qi T., Xu J. (2019). Reduced interhemispheric functional connectivity in obsessive-compulsive disorder patients. *Frontiers in Psychiatry*.

[29] Wang Y., Yin Y., Sun Y. W. (2015). Decreased prefrontal lobe interhemispheric functional connectivity in adolescents with internet gaming disorder: a primary study using resting-state FMRI. *PLoS One*.

[30] Yang T., Ren J., Li Q. (2014). Increased interhemispheric resting-state in idiopathic generalized epilepsy with generalized tonic-clonic seizures: A resting-state fMRI study. *Epilepsy Research*.

[31] Rempel-Clower N. L. (2007). Role of orbitofrontal cortex connections in emotion. *Annals of the New York Academy of Sciences*.

[32] Rolls E. (2016). A non-reward attractor theory of depression. *Neuroscience and Biobehavioral Reviews*.

[33] Ballenger J. C. (2000). Anxiety and depression: optimizing treatments. *Primary Care Companion to the Journal of Clinical Psychiatry*.

[34] Magalhaes A. C., Holmes K. D., Dale L. B. (2010). CRF receptor 1 regulates anxiety behavior via sensitization of 5-HT2 receptor signaling. *Nature Neuroscience*.

[35] Scheuerecker J., Meisenzahl E. M., Koutsouleris N. (2010). Orbitofrontal volume reductions during emotion recognition in patients with major depression. *Journal of Psychiatry & Neuroscience*.

[36] Frodl T., Bokde A. L., Scheuerecker J. (2010). Functional Connectivity Bias of the Orbitofrontal Cortex in Drug-Free Patients with Major Depression. *Biological Psychiatry*.

[37] Cheng W., Rolls E. T., Qiu J. (2016). Medial reward and lateral non-reward orbitofrontal cortex circuits change in opposite directions in depression. *Brain*.

[38] Etkin A., Prater K., Schatzberg A., Menon V., Greicius M. D. (2009). Disrupted amygdalar subregion functional connectivity and evidence of a compensatory network in generalized anxiety disorder. *Archives of General Psychiatry*.

[39] Bryant R. A., O'Donnell M. L., Creamer M., McFarlane A. C., Clark C. R., Silove D. (2010). The psychiatric sequelae of traumatic injury. *The American Journal of Psychiatry*.

[40] Bigler E., Anderson C., Blatter D. (2002). Temporal lobe morphology in normal aging and traumatic brain injury. *American Journal of Neuroradiology*.

