# Supplementary material for: Decreased Interhemispheric Functional Connectivity and Its Associations with Clinical Correlates following Traumatic Brain Injury
Source: Biomed Res Int. 2022 Apr 7;2022:3408660. doi: 10.1155/2022/3408660 (PMC9012975; doi:10.1155/2022/3408660)
Supplement: Supplementary 1 — Supplement 1: the raw data of our research. [file 3408660.f1.pdf]

ID	Education	Head Motion	TIV	Anxiety	Depression
TBI_01	15	0.08	1455	2	1
TBI_02	15	0.07	1634.1	9	4
TBI_03	15	0.07	1556.4	2	0
TBI_04	6	0.06	1534.1	6	4
TBI_05	1	0.13	1332.6	18	16
TBI_06	6	0.09	1378.8	16	18
TBI_07	16	0.1	1424.1	8	7
TBI_08	9	0.11	1278.4	1	0
TBI_09	12	0.12	1564.3	10	6
TBI_10	8	0.12	1509.1	4	8
TBI_11	5	0.11	1404.8	5	3
TBI_12	9	0.2	1485.5	14	19
TBI_13	9	0.14	1454.5	6	0
TBI_14	2	0.14	1335.8	1	5
TBI_15	5	0.14	1340.1	11	13
TBI_16	9	0.16	1509.4	5	2
TBI_17	2	0.18	1553.9	6	6
TBI_18	9	0.1	1729.6	7	10
TBI_19	16	0.04	1443.8	4	1
TBI_20	12	0.19	1586.8	5	3
TBI_21	9	0.06	1520.9	13	9
TBI_22	5	0.2	1454.8	14	11
TBI_23	5	0.17	1649.2	12	15
TBI_24	16	0.07	1530.3	7	9
TBI_25	9	0.16	1387.7	12	6
TBI_26	9	0.1	1333.1	7	5
TBI_27	9	0.1	1757.4	6	10
TBI_28	12	0.06	1639.4	17	7
TBI_29	12	0.11	1486.9	5	10
TBI_30	9	0.13	1331.3	14	21
HC_01	15	0.1	1667.2	6	4
HC_02	9	0.13	1622	4	5
HC_03	16	0.09	1502.8	2	0
HC_04	9	0.09	1545	4	3
HC_05	9	0.04	1426.6	7	4
HC_06	9	0.17	1291.5	2	5
HC_07	9	0.18	1553.2	3	6
HC_08	2	0.2	1405.2	4	1
HC_09	16	0.1	1645.6	4	2
HC_10	9	0.12	1449.3	6	2
HC_11	9	0.08	1392	7	2
HC_12	9	0.08	1621.7	0	0
HC_13	2	0.07	1708.8	3	0
HC_14	8	0.18	1608.6	7	1
HC_15	12	0.07	1605.3	1	4
HC_16	12	0.04	1317.8	5	3
HC_17	5	0.1	1629	11	6
HC_18	9	0.09	1426.1	6	7
HC_19	5	0.2	1343.9	3	1
HC_20	9	0.1	1637.2	3	7
HC_21	9	0.17	1630.8	3	2
HC_22	16	0.08	1920.9	4	1

Time since injury	GCS	GOSE	Intracerebral lesion	LOC	Cause of injury
6	13	7	Yes	Yes	Traffic accident
17	15	8	No	Yes	Fall
6	15	8	Yes	unknown	Traffic accident
11	13	7	Yes	unknown	Struck by/against
6	14	4	Yes	No	Traffic accident
2	14	5	Yes	Yes	Traffic accident
4	15	8	No	No	Traffic accident
4	7	4	Yes	Yes	Traffic accident
5	15	7	No	No	Struck by/against
10	15	7	No	No	Struck by/against
6	8	4	Yes	Yes	Traffic accident
11	12	4	Yes	Yes	Traffic accident
8	12	7	Yes	Yes	Traffic accident
3	12	5	Yes	Yes	Fall
4	7	5	Yes	Yes	Fall
25	8	7	Yes	Yes	Traffic accident
2	9	5	Yes	Yes	Fall
17	15	7	No	No	Traffic accident
7	14	8	Yes	Yes	Traffic accident
5	11	8	No	No	Traffic accident
2	15	5	Yes	No	Traffic accident
5	13	6	Yes	Yes	Traffic accident
4	6	5	Yes	Yes	Fall
27	15	7	Yes	Yes	Traffic accident
1	14	5	Yes	No	Traffic accident
4	14	6	Yes	Yes	Fall
15	14	5	Yes	Yes	Traffic accident
24	14	7	Yes	Yes	Struck by/against
4	14	8	Yes	Yes	Fall
12	14	5	No	No	Traffic accident
\	\	\	\	\	\
\	\	\	\	\	\
\	\	\	\	\	\
\	\	\	\	\	\
\	\	\	\	\	\
\	\	\	\	\	\
\	\	\	\	\	\
\	\	\	\	\	\
\	\	\	\	\	\
\	\	\	\	\	\
\	\	\	\	\	\
\	\	\	\	\	\
\	\	\	\	\	\
\	\	\	\	\	\
\	\	\	\	\	\
\	\	\	\	\	\
\	\	\	\	\	\
\	\	\	\	\	\
\	\	\	\	\	\
\	\	\	\	\	\
\	\	\	\	\	\
\	\	\	\	\	\

L_MidFroGy_VBM	R_MidFroGy_VBM	L_MidTemGy_VBM	R_MidTemGy_VBM	L_Frontal_Mid_Orb_FC	R_Frontal_Mid_Orb_FC
17.73	19.72	12.12	12.89	0.17	0.19
19.36	19.91	15.85	15.61	0.41	0.43
19.98	17.94	13.53	13.79	0.39	0.41
18.29	19.51	12.63	13.28	-0.03	0.06
14.17	14.86	12.18	12.07	0.36	0.4
13.63	13.97	10.75	11.43	0.2	0.23
18.29	16.78	15.16	15.33	0.42	0.45
12.83	13.89	6.47	10.9	0.26	0.33
18.24	16.27	13.84	14.37	0.42	0.42
17.21	16.86	13.78	15.09	0.26	0.28
16.7	15.71	12.3	13.24	0.1	0.14
8.05	19.93	9.42	15.38	0.42	0.39
16.55	16.99	10.6	12.52	0.19	0.2
13.32	13.15	10.11	11.48	0.15	0.1
15.4	13.97	11.43	10.86	0.26	0.25
17.43	7.45	12.33	13.1	0.03	0.11
14.4	14.24	11.69	12.35	0.29	0.26
21.84	21.39	15.04	15.22	0.41	0.42
16.54	16.41	10.81	11.82	0.36	0.37
18.03	16.63	15.45	15.75	0.46	0.46
18.48	16.68	13.37	15.32	0.46	0.42
13.62	19.61	11.58	10.39	0.16	0.14
16.32	17.01	11.67	11.96	0.56	0.58
19.47	18.19	12.57	14.1	0.44	0.46
14.26	15.14	12.36	13.83	0.6	0.7
16.31	16.25	11.97	11.49	0.44	0.42
23.26	25.95	15.68	18.35	0.63	0.6
18.23	19.06	13.61	13.73	0.47	0.46
17.39	16.82	13.14	12.51	0.48	0.45
16.84	16.75	13.81	14.2	0.61	0.61
21.86	22.06	16.48	16.02	0.63	0.65
22.38	22.07	15.39	16.41	0.53	0.52
15.81	16.19	12.24	13.75	0.43	0.47
17.72	17.92	13.34	14.7	0.81	0.83
20.21	18.45	12.35	12.77	0.78	0.81
14.68	16.33	11.87	11.82	1.08	1.16
16.35	18.14	12.87	14.62	0.79	0.82
15.77	16.15	12.75	12.2	0.63	0.71
19.45	20.95	14.45	13.57	0.69	0.68
16.66	17.12	12.16	13.06	0.59	0.59
14.97	15.1	13.16	11.54	0.53	0.54
18.43	20.68	14.79	15.07	0.58	0.63
19.15	19.89	15.37	15.7	0.73	0.74
20.32	19.52	16.18	16.54	0.78	0.81
21.15	20.84	15.11	15.78	0.49	0.5
15.91	16.52	12.37	12.75	0.6	0.58
21.69	21.69	17.52	16.05	0.68	0.7
17.4	17.05	13.22	14.6	0.99	1.01
15.66	15.39	12.73	12.88	0.49	0.47
17.58	18.98	14.29	14.4	0.7	0.7
18.67	18.58	13.64	14.06	0.47	0.5
23.63	23.94	17.93	17.87	0.45	0.5

L_Temoral _Mid_FC	R_Temoral _Mid_FC	pcc_FA	mcc_FA	acc_FA
0.38	0.36	0.685	0.583	0.518806
0.42	0.48	0.6732	0.558	0.513717
0.59	0.64	0.6922	0.56	0.517822
0.43	0.44	0.6575	0.535	0.443839
0.6	0.61	0.6904	0.604	0.536048
0.28	0.41	0.6776	0.544	0.504433
0.51	0.49	0.6771	0.57	0.520752
0.12	0.07	0.615	0.532	0.473379
0.41	0.41	0.6976	0.589	0.547117
0.29	0.32	0.698	0.582	0.537361
0.1	0.13	0.6471	0.534	0.490778
0.06	0.1	0.6911	0.602	0.525392
0.58	0.58	0.6704	0.584	0.511329
0.32	0.38	0.6777	0.531	0.51165
0.34	0.4	0.6392	0.513	0.453801
0.59	0.59	0.6392	0.534	0.476529
0.75	0.82	0.7177	0.618	0.545526
0.32	0.46	0.6653	0.586	0.519662
0.39	0.39	0.6974	0.607	0.556411
0.28	0.28	0.6609	0.577	0.517703
0.46	0.45	0.6825	0.633	0.549532
0.18	0.21	0.6603	0.545	0.397023
0.3	0.29	0.6509	0.564	0.514586
0.18	0.19	0.6814	0.568	0.521151
0.29	0.38	0.6903	0.583	0.524574
0.19	0.2	0.6853	0.591	0.511577
0.04	0	0.7093	0.589	0.572165
0.51	0.53	0.6818	0.576	0.508848
0.49	0.56	0.688	0.586	0.533666
0.38	0.45	0.7008	0.593	0.517377
0.6	0.66	0.6948	0.634	0.55803
0.66	0.71	0.6998	0.613	0.544135
0.7	0.71	0.7237	0.656	0.590228
0.59	0.6	0.7182	0.638	0.57546
0.49	0.51	0.6937	0.58	0.533831
0.78	0.74	0.6732	0.602	0.533382
0.8	0.85	0.6893	0.619	0.548846
0.81	0.8	0.7076	0.587	0.553767
0.65	0.69	0.6959	0.584	0.545993
0.79	0.78	0.7318	0.612	0.573029
0.61	0.65	0.6986	0.59	0.566262
1.07	1.1	0.6758	0.537	0.511563
0.63	0.74	0.6938	0.586	0.548751
0.42	0.5	0.6886	0.617	0.53842
0.64	0.63	0.6758	0.578	0.530425
0.34	0.39	0.6824	0.572	0.526686
0.76	0.84	0.7074	0.628	0.564704
0.73	0.79	0.6985	0.596	0.54614
0.35	0.34	0.7197	0.625	0.549638
0.8	0.78	0.722	0.637	0.580797
0.49	0.57	0.657	0.584	0.524037
0.64	0.65	0.715	0.652	0.568981
